# Practical NicE-seq workflow for chromatin accessibility analysis in plants

**DOI:** 10.1016/j.mocell.2026.100312

**Published:** 2026-01-06

**Authors:** Zein Eddin Bader, Nassem Albakri, Dae-Jin Yun, Young Hun Song, Junghoon Park

**Affiliations:** 1 Reproductive System RIKEN ECL Research Team, RIKEN Center for Sustainable Resource Science, Yokohama, Japan; 2 Department of Advanced Biotechnology, Konkuk University, 05029, Seoul, Korea; 3 Global Plant Stress Research Center, Konkuk University, 05029, Seoul, Korea; 4 Department of Agricultural Biotechnology, Seoul National University, Seoul 08826, Korea; 5 Research Institute of Agricultural Life Sciences, Seoul National University, Seoul 08826, Korea; 6 Plant Genomics and Breeding Institute, Seoul National University, Seoul 08826, Korea

**Keywords:** Analysis, ATAC-seq, Chromatin, NicE-seq

## Abstract

Open chromatin profiling identifies regulatory DNA regions that are accessible to transcription factors and other proteins, offering insights into gene regulation. Although ATAC-seq is commonly used for mapping open chromatin, standard techniques such as DNase-seq and ATAC-seq have limitations, including the need for large cell numbers or fresh (unfixed) samples. NicE-seq offers an alternative approach by using nicking endonucleases combined with polymerase–mediated biotin labeling. Here, we present a detailed analysis framework for NicE-seq data in plants using *Arabidopsis thaliana* as our reference species, adapted from the nf-core/atacseq pipeline with specific modifications. We emphasize the analytical differences between NicE-seq and ATAC-seq, describe data processing workflows, and illustrate methods for peak calling, annotation, and integration with transcriptomic data. This computational resource aims to guide researchers in applying NicE-seq, providing a basis for selecting between NicE-seq and ATAC-seq in plant epigenomic research, especially when working with challenging samples such as archived tissues or small cell populations.

## INTRODUCTION

**Fig. 1:**
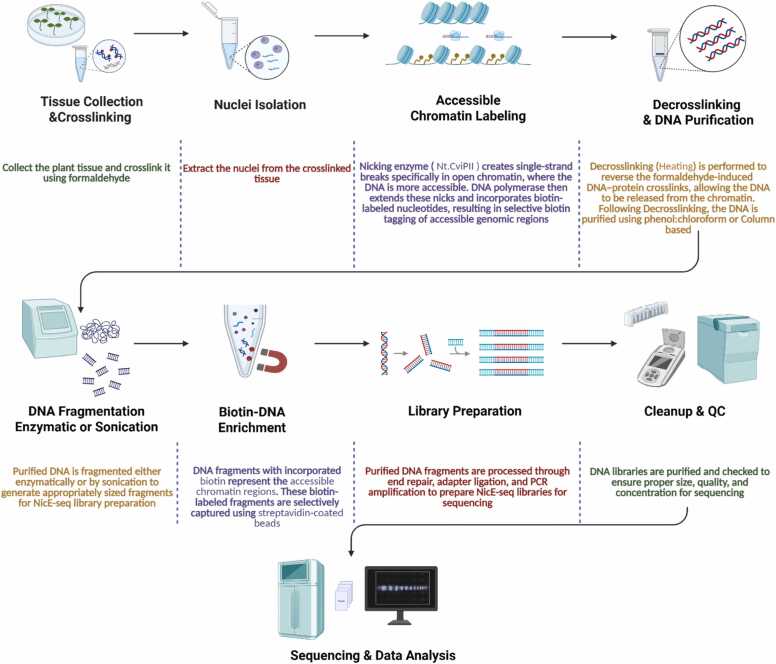
NicE-seq experimental workflow. This schematic provides an overview of the NicE-seq protocol, summarizing each major experimental step, from plant tissue collection and crosslinking to nuclei isolation, labeling, DNA purification, DNA fragmentation, Biotin enrichment, library preparation, QC, and sequencing.

In eukaryotes, DNA is packaged into chromatin, but specific regions remain nucleosome-free and accessible, functioning as regulatory elements essential for controlling transcription (Tsompana and Buck, 2014). Mapping these open chromatin regions across the genome provides valuable insights into gene regulation (Boyle et al., 2008). Established methods for profiling chromatin accessibility include DNase I hypersensitive site sequencing (DNase-seq) (Song and Crawford, 2010) and the Assay for Transposase-Accessible Chromatin (ATAC-seq) (Buenrostro et al., 2015). DNase-seq identifies accessible regions through the preferential cleavage of nucleosome-free DNA by DNase I; however, this approach typically requires large numbers of cells and careful experimental optimization, such as enzyme titration (Song and Crawford, 2010). ATAC-seq, by contrast, employs a hyperactive Tn5 transposase to insert sequencing adapters into accessible DNA, offering greater sensitivity and speed. However, ATAC-seq works best with fresh, unfixed nuclei and is often affected by high levels of mitochondrial DNA contamination, with up to half of the reads deriving from mitochondrial genomes (Grandi et al., 2022, Buenrostro et al., 2015). Consequently, both DNase-seq and ATAC-seq are suboptimal for analyzing fixed tissues or very limited cell populations, which restricts their broader application. NicE-seq (Nicking Enzyme assisted sequencing), first introduced in mammalian systems (Ponnaluri et al., 2017), offers an alternative strategy by utilizing nicking endonucleases coupled with polymerase–mediated biotin labeling, thereby improving sensitivity and reducing background noise. Despite these advantages, applications of this method in plant studies have been limited, and the experimental workflow presented in Figure 1 was derived from an established protocol (Ponnaluri et al., 2017). In this study, we describe an adapted NicE-seq analysis workflow for plants using *Arabidopsis thaliana*, derived from the nf-core/atacseq pipeline (Grandi et al., 2022) and demonstrate the critical differences between NicE-seq and ATAC-seq analyses.

## COMPUTATIONAL ANALYSIS PIPELINE

**Fig. 2:**
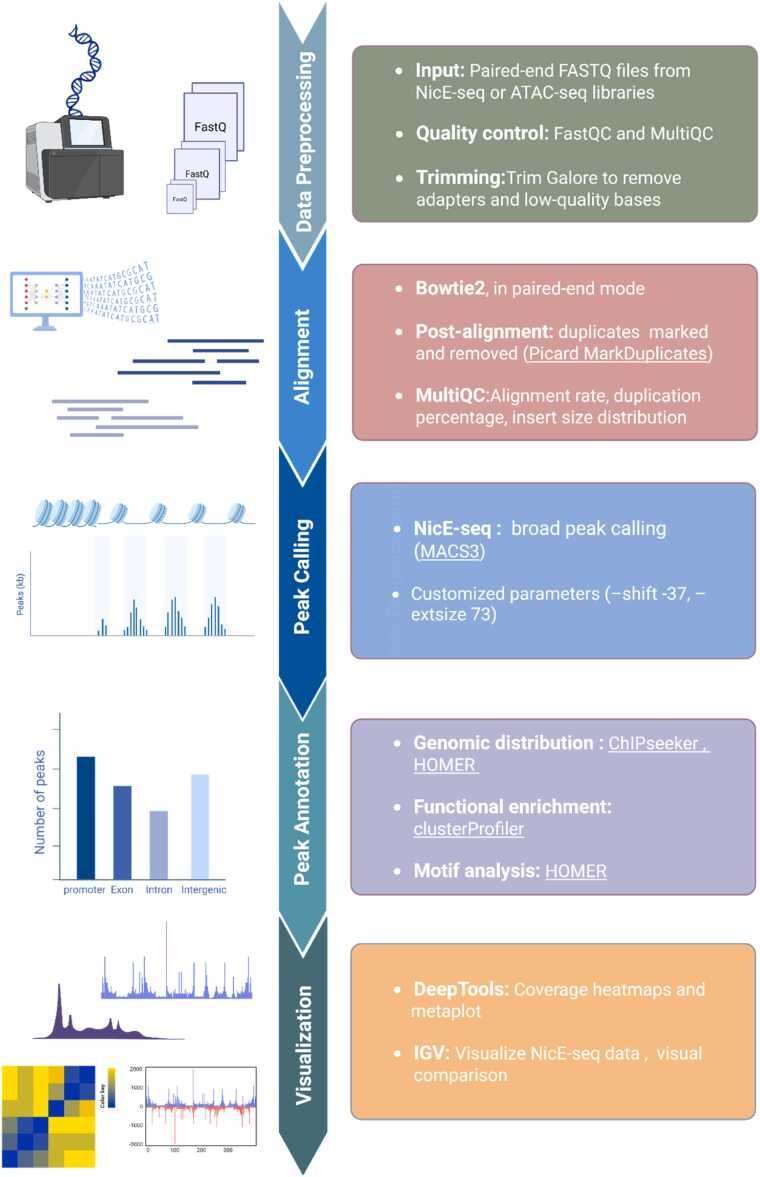
Overview of the NicE-seq computational analysis pipeline. The workflow begins with preprocessing, which includes quality control and adapter trimming, followed by the alignment of reads to the TAIR10 reference genome for *A thaliana*. Next, peak calling is performed to identify accessible chromatin regions, with subsequent peak annotation to associate peaks with genomic features such as promoters, gene bodies, and intergenic regions. Finally, results are visualized through genome browser tracks (eg, IGV, deepTool).

Genome–wide open chromatin sites were identified using the nf-core/atacseq pipeline with specific modifications that optimized for NicE-seq. The pipeline consists of the following steps: data preprocessing, alignment, peak calling, peak annotation, and visualization (Fig. 2).

## DATA PREPROCESSING

FASTQ files should be checked with FastQC or MultiQC and trimmed for adapters or low-quality bases if needed. Paired–end ATAC data may require extra trimming of short reads, while NicE-seq fragments are variable, so standard QC is sufficient.

• *Input:* Paired–end FASTQ files from NicE-seq or ATAC-seq libraries
• *Quality control:* FastQC and MultiQC
• *Trimming:* Trim Galore to remove adapters and low-quality bases (Krueger, 2015)

## ALIGNMENT

Sequencing reads were aligned to the *A thaliana* reference genome (TAIR10) using the short–read aligner Bowtie2 (Langmead and Salzberg, 2012) in paired-end mode, with stringent filters applied to maintain high mapping quality. A typical command was:

bowtie2 -x TAIR10_index -1 sample_R1.fastq.gz -2 sample_R2.fastq.gz \

--very-sensitive \
-X 2000 \
--no-mixed --no-discordant \
-p 8 \
| samtools view -bS -q 30 -F 4 -F 8 -o sample.bam

*Parameters explained*:

• --very-sensitive: maximizes alignment sensitivity
• -X 2000: sets max fragment length (2,000 bp)
• --no-mixed --no-discordant: excludes improper pairs
• -p 8: runs on 8 threads
• samtools view -q 30: keeps reads with MAPQ ≥30
• -F 4 -F 8: removes unmapped or improperly paired reads

Postalignment, duplicates were marked and removed with Picard MarkDuplicates (Zhao, 2018) using the following command:

picard MarkDuplicates I=sample.bam O=sample.dedup.bam M=sample.metrics.txt REMOVE_DUPLICATES=true VALIDATION_STRINGENCY=LENIENT

The resulting BAM file was then indexed with Samtools (Ramirez-Gonzalez et al., 2012):

samtools index sample.dedup.bam

QC metrics included alignment rate, duplication percentage, insert size distribution, and duplication summary metrics reported by Picard. These QC reports were compiled using MultiQC for integrative visualization.

● *Filtering:* Removal of low-quality reads and duplicates was performed using Samtools and Picard. Reads with mapping quality below 30 were filtered using:
  samtools view -b -q 30 sample.bam > sample.filtered.bam

Duplicates were then marked and removed with Picard:

picard MarkDuplicates I=sample.filtered.bam O=sample.dedup.bam M=sample.metrics.txt REMOVE_DUPLICATES=true VALIDATION_STRINGENCY=LENIENT

This 2-step process ensured that only high-confidence, nonredundant reads were retained for downstream analysis.

• *QC metrics:* Alignment rate, duplication levels, and insert size distributions were summarized using MultiQC. A typical command was:
  multiqc./ --outdir qc_reports

This aggregates FastQC, Bowtie2, Samtools, and Picard outputs into a single interactive report. Alignment statistics were obtained using:

samtools flagstat sample.dedup.bam > sample.flagstat.txt

Insert size distributions were generated with:

picard CollectInsertSizeMetrics I=sample.dedup.bam O=insert_size_metrics.txt H=insert_size_histogram.pdf M=0.5

Duplication levels were confirmed from the Picard MarkDuplicates output metrics file.

## PEAK CALLING

Peak calling is a critical step where the analytical differences between ATAC-seq and NicE-seq become most evident. For ATAC-seq, MACS2 is commonly used in narrow peak mode with default shift and extension parameters (--shift -37, --extsize 73) (Zhang et al., 2008), optimized for Tn5-mediated cuts. A typical command was:

macs2 callpeak -t sample.dedup.bam -f BAMPE -g 1.35e8 -n atac_seq --outdir macs2_results \

--nomodel --shift -37 --extsize 73 -q 0.01

In contrast, NicE-seq benefits from broad peak calling due to the extended labeling pattern introduced by nicking and polymerase incorporation. We recommend MACS2 or MACS3 in broad mode with custom parameters:

macs3 callpeak -t sample.dedup.bam -c input.bam -f BAMPE -n nice_seq --outdir macs3_results \

--broad --broad-cutoff 0.1 --nomodel --shift -37 --extsize 73 -q 0.05

MACS3 is preferred for NicE-seq as it provides improved statistical modeling and more accurate boundary detection for broadly accessible domains. Including input DNA as a control is strongly recommended to minimize background and enhance specificity.

*Note on effective genome size (-g parameter)*: In MACS2, the -g parameter specifies the adequate genome size, which helps the software model background noise. For *A thaliana,* which has a compact genome (∼135 Mb), a commonly used value is 1.35e8, reflecting the mappable portion of the genome. While MACS3 can automatically infer adequate genome size when -g is omitted, explicitly setting -g is still recommended for consistency, reproducibility, and comparability across analyses. If omitted, MACS3 estimates it based on the genome assembly and read coverage, but this may vary slightly depending on dataset characteristics.

• *NicE-seq:* Broad peak calling with customized parameters (--shift -37, --extsize 73) reflecting Nt.CviPII fragment architecture
• *Input control:* Input DNA or nonlabeled DNA used to refine peak specificity

## PEAK ANNOTATION

Peak annotation was carried out using ChIPseeker (Yu et al., 2015) and HOMER to characterize the genomic distribution of identified peaks. The following command was used in R with ChIPseeker:

library(ChIPseeker)
library(TxDb.Athaliana.BioMart.plantsmart28)

peak <- readPeakFile("macs3_results/nice_seq_peaks.broadPeak")
txdb <- TxDb.Athaliana.BioMart.plantsmart28
peakAnno <- annotatePeak(peak, tssRegion=c(-2000, 2000), TxDb=txdb)
plotAnnoPie(peakAnno)

This analysis classified peaks into promoters (±2 kb from TSS), exons, introns, and intergenic regions. To identify functional enrichment, clusterProfiler (Yu et al., 2012) was used:

library(clusterProfiler)
gene <- as.data.frame(peakAnno)$geneId
enrichGO(gene = gene,
  OrgDb = org.At.tair.db,
  keyType = "TAIR",
  ont = "BP",
  pAdjustMethod= "BH",
  pvalueCutoff = 0.05,
  qvalueCutoff = 0.2)

In parallel, HOMER was used for motif analysis:

findMotifsGenome.pl macs3_results/nice_seq_peaks.broadPeak TAIR10.fa homer_output/ -size given

This provided motif enrichment for transcription factors potentially regulating accessible regions.

## VISUALIZATION

● *deepTools:* Coverage heatmaps and metaplots were generated using the following commands (Ramírez et al., 2014). First, normalized bigWig files were created:
  bamCoverage -b sample.dedup.bam -o sample.bw --normalizeUsing RPGC --effectiveGenomeSize 1.1948e8 \
  --binSize 10 -p 8

Coverage heatmaps were produced with:

computeMatrix scale-regions -S sample.bw -R genes.bed \
--beforeRegionStartLength 2000 --regionBodyLength 5000 --afterRegionStartLength 2000 \
--skipZeros -o matrix.gz -p 8

Heatmaps and metaplots were then visualized using:

plotHeatmap -m matrix.gz -out heatmap.pdf
plotProfile -m matrix.gz -out profile.pdf

• *IGV* (Robinson, 2017): can be used to visualize NicE-seq data, showing signal tracks relative to genes. Typical snapshots reveal enrichment at promoters or regulatory sites and reduced signal across nucleosome-covered gene bodies, similar to ATAC-seq patterns. Browser tracks comparing NicE-seq and ATAC-seq accessibility profiles were visualized using normalized bigWig files. Normalization was performed with deepTools as follows:
  bamCoverage -b sample.dedup.bam -o sample.normalized.bw \.
  --normalizeUsing RPGC --effectiveGenomeSize 1.20e8 \.
  --binSize 10 -p 8.

This process produces genome–wide coverage files in bigWig format, normalized by reads per genomic content (RPGC). The bigWig outputs from both NicE-seq and ATAC-seq can be visualized in the Integrative Genomics Viewer (IGV) to compare accessibility profiles across regions of interest directly. All tracks are aligned to the TAIR10 genome assembly, with uniform scaling applied to ensure accurate comparisons between conditions.

## NICE-SEQ VS ATAC-SEQ: KEY DIFFERENCES

**Fig. 3:**
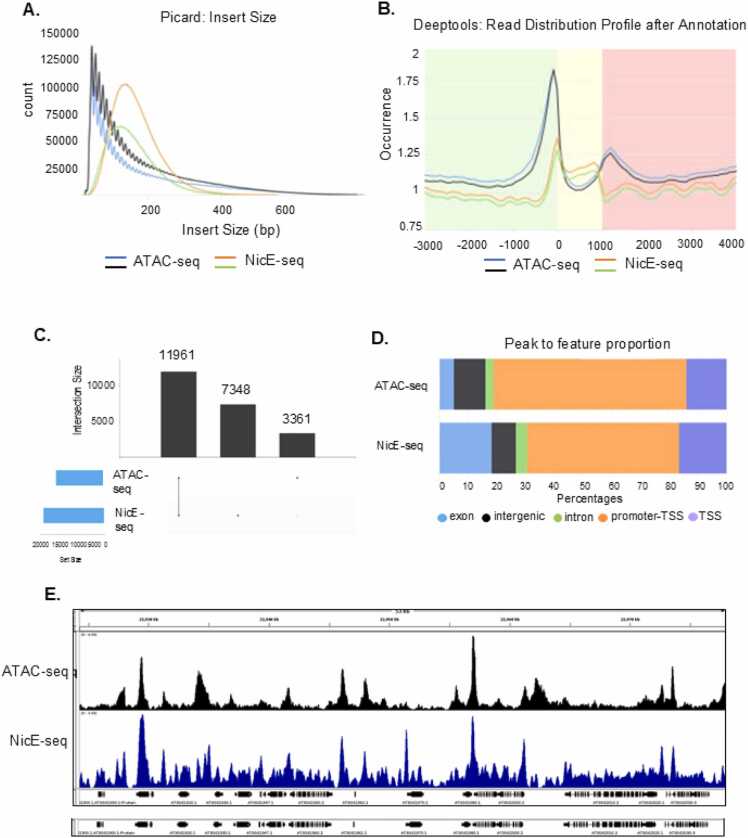
Comparison between NicE-seq and ATAC-seq. (A) Scaled insert size distributions for NicE-seq (black and blue) and ATAC-seq (orange and green) libraries across biological replicates under normal conditions. (B) Average NicE-seq and ATAC-seq signal profiles across transcription start sites (TSS; −3,000 to +4,000 bp). (C) Counts of consensus and unique peaks identified by each method. (D) Proportions of peaks annotated to different genomic features. (E) IGV snapshot of a 53 kb genomic region illustrating accessible chromatin peaks detected by NicE-seq and ATAC-seq.

Analytical differences between ATAC-seq and NicE-seq:

In computational analysis, ATAC-seq and NicE-seq follow similar preprocessing steps, such as quality assessment, adapter trimming, and alignment to the reference genome. The significant distinctions arise during peak calling and data interpretation. ATAC-seq commonly uses narrow peak calling with MACS2, which is well-suited for detecting transcription factor binding sites and sharp regions of accessible chromatin. By contrast, NicE-seq is better analyzed with broad peak calling, since its biotin-labeling and nicking enzyme strategy identifies extended accessible regions, often covering promoters and regulatory domains rather than precise motifs. Additionally, NicE-seq libraries typically exhibit distinct fragment length distributions and show stronger peak enrichment downstream of the TSS region (Fig. 3A and B), requiring customized parameters during peak calling (--shift -37, --extsize 73). These analytical choices strongly influence downstream annotation. NicE-seq peaks are often enriched at promoters and across gene bodies, while ATAC-seq peaks are more likely to mark discrete transcription factor binding sites (Fig. 3C and D). The IGV snapshot further illustrates that accessible chromatin peaks within the gene body are more prominent in NicE-seq compared to ATAC-seq (Fig. 3E). Consequently, visualization and integrative analyses must be tailored to each method’s resolution and inherent biases.

## CONCLUSION

We present a NicE-seq computational pipeline adapted from nf-core/atacseq, alongside a direct comparison with ATAC-seq. This resource provides plant researchers with guidance in choosing the most suitable chromatin accessibility method based on tissue type, experimental goals, and resource availability. Our integrated framework paves the way for broader adoption of NicE-seq in plant genomics, offering new opportunities to uncover regulatory mechanisms underlying development and stress responses in *Arabidopsis*.

## Data Availability Statement

All data supporting the findings of this study are available in the paper.

## Funding and Support

This work was supported by the 10.13039/501100003725National Research Foundation of Korea (NRF) grants funded by the Korea government Ministry of Science and ICT (MSIT) (RS-2024-00407469 to D.-J.Y. and J.P., 2022R1A2C3004098 to D.-J.Y., RS-2025-00562751 to Y.H.S. RS-2023-00239735 to J.P.).

## Author Contributions

**Nassem Albakri:** Writing – original draft, Formal analysis, Data curation, Conceptualization. **Zein Eddin Bader:** Formal analysis, Data curation, Conceptualization. **Dae-Jin Yun:** Supervision, Project administration, Funding acquisition. **Young Hun Song:** Writing – review & editing, Funding acquisition. **Junghoon Park:** Supervision, Funding acquisition, Conceptualization.

## Declaration of Competing Interest

The authors declare that they have no known competing financial interests or personal relationships that could have appeared to influence the work reported in this paper.
